# Prior observation of fear learning enhances subsequent self-experienced fear learning with an overlapping neuronal ensemble in the dorsal hippocampus

**DOI:** 10.1186/s13041-019-0443-6

**Published:** 2019-03-14

**Authors:** Hiroshi Nomura, Chie Teshirogi, Daisuke Nakayama, Masabumi Minami, Yuji Ikegaya

**Affiliations:** 1 Department of Pharmacology, Graduate School of Pharmaceutical Sciences, Hokkaido University, Nishi 6, Kita 12, Kita-ku, Sapporo, 060-0812 Japan; 2 Laboratory of Chemical Pharmacology, Graduate School of Pharmaceutical Sciences, The University of Tokyo, Tokyo, 113-0033 Japan; 3 Center for Information and Neural Networks, National Institute of Information and Communications Technology, Osaka, 565-0871 Japan

**Keywords:** Fear memory, Memory engram, Observational learning, CA1, Mirror neuron, Social behavior, Communication

## Abstract

Information from direct experience and observation of others is integrated in the brain to enable appropriate responses to environmental stimuli. Fear memory can be acquired by observing a conspecific’s distress. However, it remains unclear how prior fear observation affects self-experienced fear learning. In this study, we tested whether prior observation of a conspecific receiving contextual fear conditioning affects subsequent self-experienced fear conditioning and how neuronal ensembles represent the integration of the observation and self-experience. Test mice observed demonstrator mice experiencing fear conditioning on day 1 and directly experienced fear conditioning on day 2. Contextual fear memory was tested on day 3. The prior observation of fear conditioning promoted subsequent self-experienced fear conditioning in a hippocampus-dependent manner. We visualized hippocampal neurons that were activated during the observation and self-experience of fear conditioning and found that self-experienced fear conditioning preferentially activated dorsal CA1 neurons that were activated during the observation. When mice observed and directly experienced fear conditioning in different contexts, preferential reactivation was not observed in the CA1, and fear memory was not enhanced. These findings indicate that dorsal CA1 neuronal ensembles that were activated during both the observation and self-experience of fear learning are implicated in the integration of observation and self-experience for strengthening fear memory.

## Introduction

Humans and animals acquire memories through direct experience and observation of others. Information from both these sources is integrated in the brain to enable appropriate responses to environmental stimuli. Fear memory can be socially acquired from vicarious observation in humans and animals [1, 2]. Mice that observed other mice experiencing fear conditioning display freezing behavior when subsequently exposed to the conditioned stimulus [3]. Visual, olfactory, and auditory cues contribute to the development of fear conditioning [3, 4]. The majority of previous studies have focused on observational fear learning itself and showed the neural circuit mechanisms of observational fear learning. For example, the anterior cingulate cortex and basolateral amygdala are involved in observational fear learning [4–7]. However, memories from observation and direct experience may influence each other to refine memories. In this regard, it is poorly understood how prior fear observation affects subsequent self-experienced fear learning. Specifically, neuronal activity during the integration of information from prior observation and subsequent self-experience is unclear.

Subsets of neurons in memory-related areas contribute to memory acquisition, consolidation, and retrieval [8–12]. Neurons that are activated during memory acquisition are involved in consolidation and retrieval of the memory [13–15]. When a subject learns an association between two stimuli, certain subsets of neurons receive convergent information regarding both stimuli. For example, subpopulations of neurons in the amygdala and frontal association cortex preferentially receive convergent information regarding context and shock during contextual fear conditioning [16, 17]. Neuronal ensembles receiving multiple inputs are necessary for integrating information [18]. We therefore hypothesized that a subset of neurons is preferentially activated by both observation and self-experience of fear conditioning and that this overlapping ensemble is associated with the integration of observation and self-experience.

In this study, we examined whether prior observation of fear learning affected subsequent self-experienced fear learning. Moreover, we tested whether dorsal hippocampal CA1 neurons that were activated by observation were reactivated by subsequent self-experienced fear learning. We propose a neuronal circuit mechanism that integrates observation and self-experience of fear learning.

## Results

### Prior observation of fear conditioning enhances subsequent self-experienced learning of contextual fear

**Fig. 1:**
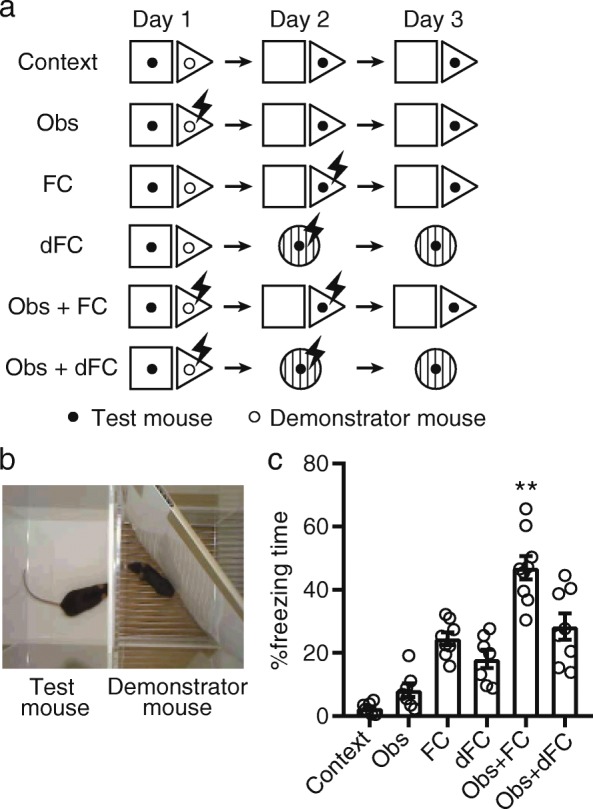
Prior observation of fear conditioning enhances subsequent self-experienced learning of contextual fear. **a** Experimental procedures for behavioral experiments. **b** A representative photo showing observation of fear conditioning. **c** Prior observation of fear conditioning enhanced subsequent learning of contextual fear. Enhancement of freezing only occurred when the test mice received fear conditioning in the same context where the demonstrator mice received fear conditioning. Context: *n* = 7, Obs: *n* = 7, FC: *n* = 8, dFC: *n* = 7, Obs + FC: *n* = 9, Obs + dFC: *n* = 8. Tukey’s test after one-way ANOVA, *F*_5,40_ = 29.59, *P* < 0.0001. **P < 0.01 vs. Obs + FC vs. other groups. Values are reported as mean ± SEM

To examine the effect of prior observation of fear conditioning on self-experienced learning of contextual fear, test mice observed demonstrator mice receiving fear conditioning on day 1 and directly experienced fear conditioning on day 2. Contextual fear memory was tested on day 3 (Fig. 1a, Obs + FC group). For observation of fear conditioning, the test mouse was exposed to an observation chamber adjacent to a conditioning chamber in which the demonstrator mouse received shocks. The two chambers were partitioned by transparent walls (Fig. 1b). On day 2, the test mice received a footshock in the conditioning chamber. On day 3, test mice were re-exposed to the conditioning chamber for assessment of contextual fear memory. We prepared five behavioral control groups including mice that only underwent context exposure (Context group), mice that only observed fear conditioning (Obs group), mice that only received fear conditioning (FC and dFC groups), and mice that observed and underwent fear conditioning in different contexts (Obs + dFC group) (Fig. 1a). Compared to the control groups, the Obs + FC group demonstrated longer freezing during the memory test (Fig. 1c).

### The dorsal hippocampus mediates observation-induced enhancement of fear learning

**Fig. 2:**
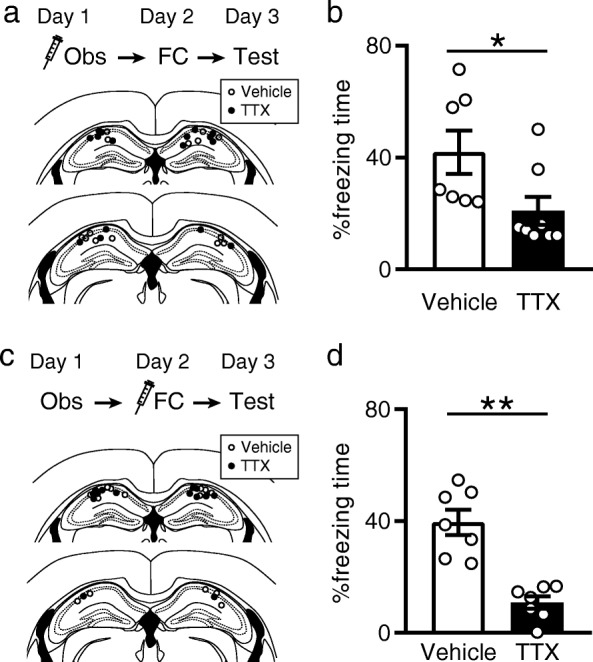
The hippocampus is involved in observation-induced enhancement of fear learning. **a** TTX was infused into the hippocampus before observation of fear conditioning on day 1. **b** TTX infusions into the hippocampus before observation of fear conditioning impaired observation-induced enhancement of fear learning. Vehicle: *n* = 7, TTX: n = 8. Student’s *t*-test, *t*_13_ = 2.3, *P = 0.037. **c** TTX was infused into the hippocampus before self-experienced fear conditioning on day 2. **d** TTX infusions into the hippocampus before self-experienced fear conditioning impaired observation-induced enhancement of fear learning. Vehicle: *n* = 7, TTX: *n* = 7. Student’s *t*-test, *t*_12_ = 5.7, *P < 0.0001. Values are reported as mean ± SEM

The dorsal hippocampus is implicated in standard contextual fear memory [19]. We thus speculated that the dorsal hippocampus may be involved in observation-induced enhancement of contextual fear learning. To inhibit hippocampal activity during the observation of fear conditioning, we bilaterally infused tetrodotoxin (TTX) into the dorsal hippocampus before the observation. The mice underwent fear conditioning on day 2, and contextual fear memory was tested on day 3. The mice exhibited shorter freezing during the memory test. (Fig. 2a). To examine the effects of inhibiting hippocampal activity when directly receiving fear conditioning, we used separate mice that observed fear conditioning on day 1 and received TTX infusions into the dorsal hippocampus before fear conditioning on day 2. TTX infusions impaired freezing behavior during the test on day 3 (Fig. 2b). Collectively, these results suggest that the dorsal hippocampus is involved in observation-induced enhancement of fear learning.

### Fear conditioning preferentially activates dorsal CA1 neurons that were activated during observation of fear conditioning

**Fig. 3:**
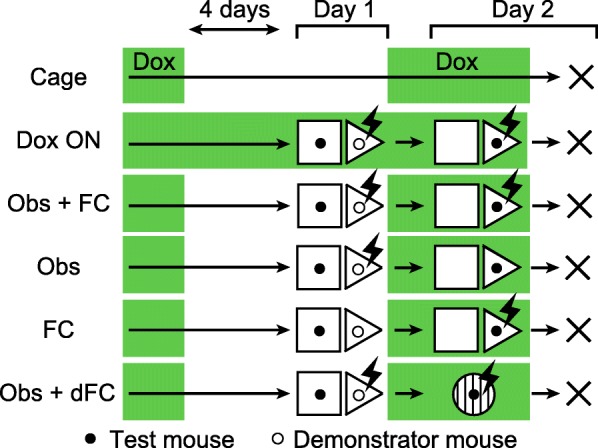
Experimental procedures for visualizing neurons activated during observation and self-experience of fear conditioning. Doxycycline was removed from the diet of Obs + FC, Obs, FC, and Obs + dFC groups 4 days before the first behavioral task. The next day, mice underwent fear conditioning or context exposure with doxycycline. The Dox ON group observed and received fear conditioning with doxycycline. Mice in the Cage group were kept in their homecages on the same schedule of doxycycline treatment to that of the Obs + FC group. Cage: *n* = 4, Dox ON: *n* = 4, Obs + FC: *n* = 5, Obs: *n* = 5, FC: *n* = 5, Obs + dFC: *n* = 5

**Fig. 4:**
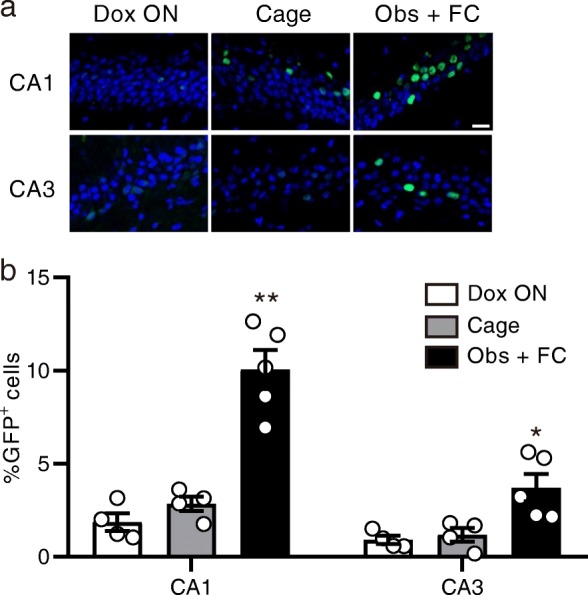
Observation of fear conditioning enhances the percentage of GFP^+^ cells in the hippocampal CA1 and CA3 in a doxycycline-dependent manner. **a** Representative images of GFP immunostaining in the CA1. The scale bar represents 30 μm. **b** Percentage of GFP^+^ cells in the CA1 and CA3. Tukey’s test after two-way ANOVA, group effect: *F*_2, 20_ = 41.95, *P* < 0.0001. **P < 0.05, **P < 0.01 vs. Dox ON group. Values are reported as mean ± SEM

To label the neurons that were activated during the observation of fear conditioning, we used Fos-H2BGFP mice. In these transgenic mice, tetracycline-transactivator (tTA) is produced by activation of the activity-dependent c-Fos promoter. Without doxycycline, tTA binds to tetracycline operator (tetO) sequence and triggers the expression of human histone H2B-GFP protein in activated neurons. The mice were raised with doxycycline, which was removed 4 d before starting behavioral experiments. The Obs + FC group observed fear conditioning and were then administered a high dose of doxycycline (1 g/kg) for rapid inhibition of H2B-GFP expression. The next day, they received fear conditioning and were perfused 2 h later (Fig. 3). The Cage group did not undergo a behavioral task, but the schedule of doxycycline treatment was the same as that for the Obs + FC group. The Dox ON group observed fear conditioning and underwent fear conditioning with doxycycline. The observation of fear conditioning increased the percentage of GFP^+^ cells in the dorsal hippocampal CA1 and CA3 in a doxycycline-dependent manner (Fig. 4).

**Fig. 5:**
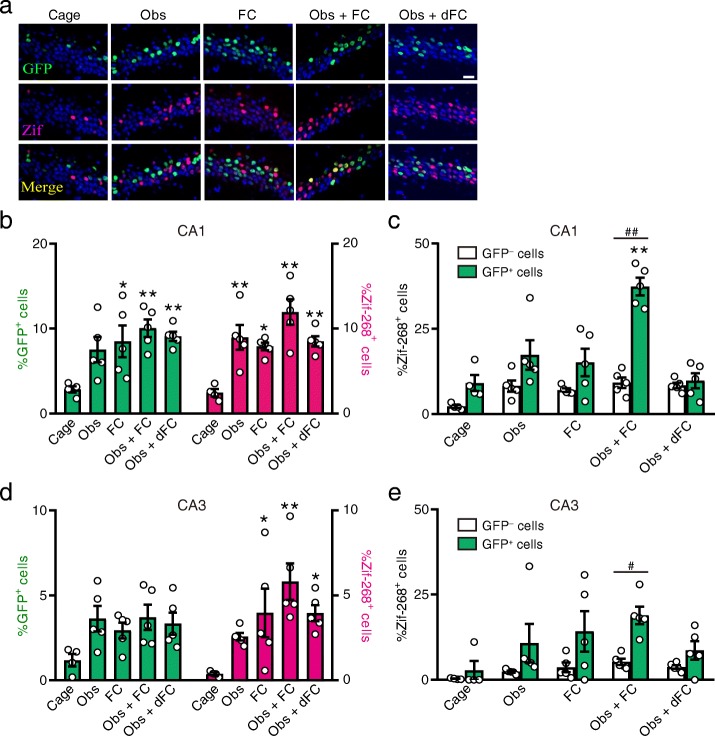
Self-experienced fear conditioning preferentially activates CA1 neurons that were activated during observation of fear conditioning. **a** Representative images of GFP and Zif-268 immunostaining in the CA1. The scale bar represents 30 μm. **b** The percentage of GFP^+^ cells and Zif-268^+^ cells in the CA1. Tukey’s test after two-way ANOVA, group effect: *F*_4, 38_ = 12.60, *P* < 0.0001. *P < 0.05, **P < 0.01 vs. Cage group. **c** The percentage of cells with Zif-268 signals in GFP^−^ and GFP^+^ cells in the CA1. Two-way ANOVA, *F*_4, 38_ = 14.21, *P* < 0.0001. ##*P* < 0.0001, GFP^−^ vs. GFP^+^. **P < 0.0001, GFP^+^ cells in Obs + FC group vs. GFP^+^ cells in other groups. **d** The percentage of GFP^+^ cells and Zif-268^+^ cells in the CA3. Tukey’s test after two-way ANOVA, group effect: *F*_4, 38_ = 6.60, *P* = 0.0004. *P < 0.05, **P < 0.01 vs. Cage group. **e** The percentage of cells with Zif-268 signals in GFP^−^ and GFP^+^ cells in the CA3. Two-way ANOVA, cell-type effect: *F*_4, 38_ = 2.87, *P* = 0.036. #*P* = 0.016, GFP^−^ vs. GFP^+^. Values are reported as mean ± SEM

To test whether the cells that were activated during the observation (GFP^+^ cells) were reactivated during fear conditioning, we prepared three additional control groups including mice that only observed fear conditioning, mice that directly received fear conditioning, and mice that both observed and directly received fear conditioning in different contexts. All mice were perfused 2 h after the second behavioral task. To identify the neurons that were activated during the second behavioral task (directly receiving fear conditioning or context exposure), we subjected brain slices to immunohistochemistry for Zif-268, a molecular marker for neuronal activity. Exposure to the context and self-experienced fear conditioning increased the percentage of Zif-268^+^ cells in the dorsal hippocampal CA1 and CA3 (Fig. 5a, b, and d). In the Obs + FC group, H2B-GFP^+^ cells preferentially expressed Zif-268 when compared to H2B-GFP^−^ cells in the CA1 (Fig. 5c). This preferential Zif-268 expression was not observed in the other control groups. The reactivation ratio, the proportion of cells with Zif-268 immunoreactivity in GFP ^+^ cells (green bars in Fig. 5c), was higher in the Obs + FC group compared to the other control groups. Importantly, the reactivation was dependent on the context because neurons that were activated by the observation were not reactivated by self-experience of fear conditioning in a different context (Obs + dFC group). In the CA3, H2B-GFP^+^ cells preferentially expressed Zif-268 when compared to H2B-GFP^−^ cells in the Obs + FC group, but the reactivation ratio was comparable across groups (Fig. 5e). Taken together, self-experienced fear conditioning preferentially activated dorsal CA1 neurons that were activated during prior observation of fear conditioning in the same context.

## Discussion

Here, we demonstrated that prior observation of fear learning enhanced subsequent self-experienced fear learning. This memory enhancement was not due to generalized fear enhancement because it did not occur when mice directly experienced fear conditioning in a different context to that of the observation. Observation-induced memory enhancement was associated with self-experience-induced reactivation of the neurons that were activated during the observation. Reactivation of these neurons may have contributed to the integration of observation and self-experience for strengthening fear memory.

Stimulus convergence in neuronal ensembles is important for learning and memory. Specific subsets of neurons receive convergent information during associative learning. For example, a subset of neurons in the amygdala and frontal association cortex are preferentially activated by both context exposure and shock during contextual fear conditioning [16, 17]. Largely overlapping neuronal ensembles activated by distinct contexts link the two contextual memories [20]. Neuronal ensembles that receive multiple inputs are necessary for integrating information. Selective inhibition of a neuronal ensemble in the amygdala that is activated by both conditioned taste aversion training and auditory fear conditioning impairs the linking of the two memories [18]. In the current study, we demonstrated that an overlapping neuronal ensemble in the dorsal hippocampal CA1 is activated by observation of fear conditioning and subsequent self-experienced fear conditioning. The overlapping neuronal ensemble was specifically associated with observation-induced memory enhancement. Indeed, activation of the overlapping neuronal population was not observed when fear memory was not enhanced in the condition whereby mice observed and directly experienced fear conditioning in different contexts. When two experiences share neuronal ensembles, neuronal plasticity (e.g. synaptic potentiation, high neuronal excitability, and structural changes) that is induced by the first experiences in neuronal ensembles [15, 21, 22] could strengthen the memory traces that are formed by the second experiences. A future study using optogenetic inhibition of the overlapping neuronal ensemble will help to elucidate whether this specific neuronal ensemble is important for integrating the observation and self-experience of fear learning.

A subset of hippocampal CA1 neurons encodes representations of both self and other, although different sensory stimuli are involved in these representations. Visual inputs are necessary for observational fear learning [4]. In addition, other sensory modalities, such as olfactory and auditory cues, may also contribute to observational learning [3, 4]. By contrast, physical pain is critical for self-experienced fear conditioning. The representations in CA1 for both self and other are not limited to observational fear learning. In an observational T-maze task in which observer rats were required to observe demonstrator rats’ trajectories to obtain a reward, a subset of CA1 neurons in observer rats had spatial receptive fields that were identical for self and other [23]. In this regard, hippocampal representations for other animals could be involved in social behavior [24, 25].

Multiple brain regions including the anterior cingulate cortex (ACC) and amygdala are implicated in observational fear learning in rodents and humans [2, 4, 6, 7, 26]. In particular, the pathway from the ACC to basolateral amygdala is activated by observational fear learning. Optogenetic inhibition of this pathway impairs observational fear learning [5]. The ACC and amygdala are also engaged in self-experience of fear conditioning. Indeed, when a brief priming footshock is followed by observation of fear conditioning, an overlapping neuronal ensemble in the ACC is activated by the priming footshock and the observation [6]. Therefore, one cannot rule out the possibility that brain regions other than the hippocampus also integrate information regarding observation and self-experience of fear conditioning. Interestingly, the CA1 cells that were activated during the observation were significantly reactivated during fear conditioning; however, the reactivation ratio in the CA3 of the mice experiencing both observation and fear conditioning was not different from control groups. The different reactivation between CA1 and CA3 could be due to the projection from the CA2. A social stimulus modifies CA2 representations of space [27]. The CA2 region is essential for social memory [25]. The CA2 region send a projection to the CA1 but not CA3 [28]. Therefore, the high reactivation in the CA1 could be due to social processing in the CA2.

In conclusion, we have demonstrated that prior observation of learning promotes subsequent self-experienced learning with an overlapping memory trace in the dorsal hippocampus. Our findings may be relevant for post-traumatic stress disorder, which emerges and is worsened by observation of a traumatic event. In addition, dysfunction in these neural substrates may underlie learning disability in autism spectrum disorder.

## Methods

### Animals

All experiments performed were approved by the animal experiment ethics committee at the University of Tokyo (approval number: 24–10) and Hokkaido University (approval number: 16–0043) according to the University of Tokyo and Hokkaido University guidelines for the care and use of laboratory animals. These experimental protocols were carried out in accordance with the Fundamental Guidelines for Proper Conduct of Animal Experiments and Related Activities in Academic Research Institutions (Ministry of Education, Culture, Sports, Science and Technology, Notice No. 71 of 2006), the Standards for Breeding and Housing of and Pain Alleviation for Experimental Animals (Ministry of Environment, Notice No. 88 of 2006), and the Guidelines on the Method of Animal Disposal (Prime Minister’s Office, Notice No. 40 of 1995).

Fos-H2BGFP mice [29, 30] were generated by crossing hemizygous transgenic mice that express tetracycline-transactivator (tTA) under the control of the *c-Fos* promoter (Jackson Laboratory, #008344) [14] with hemizygous transgenic mice that express a H2B-GFP fusion protein under the control of tetO (Jackson Laboratory, #005104). The original strain of Fos-tTA mice was (C57BL/6 × DBA/2) F2, and they were backcrossed with C57Bl/6J mice for a total of at least 8 generations in the Jaxon Laboratory and our animal facility. The original strain of tetO-H2BGFP mice was CD-1, and they were backcrossed with C57Bl/6J for a total of at least 2 generations in our animal facility. Wild-type mice were used for the experiments in Figs. 1 and 2. Mice were given free access to food and water and kept on a 12 h light/dark cycle (lights on from 6:00 A.M. to 6:00 P.M.). All mice were housed in groups of two and acclimated to daily handling for 1 week prior to the start of the study. Mice were 8–14 weeks old during behavioral experiments.

### Behavioral procedures

Contextual fear conditioning and subsequent testing were performed in a triangular conditioning chamber (length of sides: 15 cm, 18 cm, 23 cm; height: 27 cm) that had a stainless-steel grid floor and in an adjacent observation chamber (length of sides: 18 cm, 15 cm; height: 27 cm) that had an acrylic floor. The walls of the both chambers were made from acrylic plates. The two chambers were partitioned by a transparent wall. Two male cagemates were defined as either test or demonstrator mouse. During the conditioning session for observation, the demonstrator and test mice were placed in the conditioning and observation chambers, respectively. After a 5-min acclimation period, 20 shocks (1 mA, 2 s) were delivered to the demonstrator mouse through a shock scrambler (SGS-003DX; Muromachi Kikai, Tokyo, Japan) with a 12-s interval between shocks. Mice were kept in the chambers for an additional 60 s and were then returned to their home cages. Conditioning sessions for the test mouse involved placing them in the conditioning chamber and delivering 1 s footshock (0.6 mA) after 150 s. Mice were returned to their home cages after 60 s. For test sessions, mice were placed in the conditioning chamber without any shocks for 5 min. The chamber was cleaned with 70% ethanol before each session. The test session was video-recorded for automated scoring of freezing, according to a previously described method [31]. For conditioning sessions in a different context, mice received shocks in a circular chamber (diameter: 13.5 cm, height: 27 cm). The walls had vertical stripes and were covered with 1% acetic acid.

In the experiment depicted in Fig. 1, the Obs + FC group observed fear conditioning on day 1 and directly experienced fear conditioning on day 2. The Context group was exposed to the observation chamber for 10 min on day 1, when the demonstrator mouse received no shock in the conditioning chamber. They were exposed to the conditioning chamber without any shocks for 210 s on day 2. The Obs group observed fear conditioning on day 1 and was exposed to the conditioning chamber without any shocks for 210 s on day 2. The FC group was exposed to the observation chamber for 10 min on day 1, when the demonstrator mouse received no shock. They received fear conditioning on day 2. The dFC group underwent the same procedure as that of the FC group except that they received a shock in the different context on day 2. The Obs + dFC group underwent the same procedure as that of the Obs + FC group except that they received a shock in the different context on day 2. All groups underwent the test session on day 3.

In the experiment depicted in Fig. 2, mice observed fear conditioning on day 1 and directly experienced fear conditioning on day 2. They received local infusions of vehicle or TTX into the hippocampus 20 min before the observation of fear conditioning on day 1 or 20 min before direct experience of fear conditioning on day 2.

In the experiments depicted in Figs. 3, 4, and 5, mice were kept on food containing doxycycline (40 mg/kg) before behavioral experiments. Doxycycline was removed from the Obs + FC, Obs, FC, and Obs + dFC groups 4 days before the first behavioral task. Mice were given food containing 1 g/kg doxycycline after the behavioral tasks to rapidly inhibit H2B-GFP expression [13, 32]. The next day, they underwent fear conditioning or context exposure with doxycycline. The Dox ON group observed and received fear conditioning with doxycycline. All groups except for the Cage group were perfused with PBS followed by 4% PFA 2 h after the second behavioral task, and their brains subjected to immunohistochemistry. Mice in the Cage group were kept in their homecages with the same schedule of doxycycline treatment and perfusion as that of the Obs + FC group.

### Surgery

Mice were anesthetized using pentobarbital (2.5 mg/kg, i.p.) and xylazine (10 mg/kg, i.p.) followed by bilateral implantation of 26-gauge stainless-steel guide cannulas (Plastics One) in the dorsal CA1 (AP: -1.9 mm, ML: ±1.8 mm, DV: − 2.0 mm). Cannulas were secured to the skull using a mixture of acrylic and dental cement, and 33-gauge dummy cannulas were then inserted into each guide cannula to prevent clogging. Mice were allowed to recover for at least 6 d after surgery before behavioral experiments were performed.

### Drugs and microinfusions

Mice received bilateral infusions (500 nL/side) of PBS or TTX (10 μg/uL) into the hippocampus. Infusions were performed over 2 min. Infusion cannulas (28 gauge, extending 1.0 mm below the guide cannulas) were left in place for at least 1 min after infusions.

### Immunohistochemistry

After anesthetization with diethyl ether, mice were transcardially perfused with PBS followed by 4% paraformaldehyde (PFA). Brains were post-fixed in 4% PFA for 2–4 h. Free-floating coronal sections (40 μm) were prepared using a cryostat. The sections were incubated with 0.2% Triton-X-100 and 5% goat serum for 1 h, primary antibodies including polyclonal anti-Zif-268 antibody (SC-189, 1:5000; Santa Cruz) and anti-green fluorescent protein (GFP) primary antibody (A11120, 1:1000, Invitrogen) at 4 °C overnight, followed by incubation with secondary antibodies including goat anti-rabbit biotinylated antibody (A31553, 1:400; Life Technologies) and Alexa Fluor 488 goat anti-mouse IgG for 2 h, VECTASTAIN ABC Kit (Vector Laboratories) for 1.5 h, and TSA-Cyanine 3 (SAT704A001EA, 1:1000; Perkin–Elmer) for 1 h. The sections were mounted in PermaFluor (ThermoShandon, Pittsburgh, PA, United States). Nuclei were counterstained with Hoechst dye (1:1000; Invitrogen).

### Confocal microscopy and image analysis

Images of dorsal CA1 and CA3 cells (four slices per mouse) were acquired using a confocal microscope (CV1000, YOKOGAWA, Tokyo, Japan) at 20× magnification. Areas of analysis were z-sectioned in 2-μm optical sections. The same laser and scanning settings were used for images within an experiment to allow for comparison across groups. The nuclei in the CA1 and CA3 were traced in the Hoechst images using ImageJ software (NIH). Only cells that were presumptive neurons, with large nuclei stained diffusely with Hoechst, were included in the analysis. The traced regions were copied to corresponding GFP and Zif-268 images for analysis. An experimenter blind to behavioral conditions assigned labels for GFP and Zif-268. The number of CA1 and CA3 cells quantified per mouse were 596.2 ± 14.0 and 557.3 ± 12.0 cells, respectively.

### Data analysis

All values are given as the mean ± standard error of the mean (SEM). Student’s *t*-test, Tukey’s test, and Sidak’s test after analysis of variance (ANOVA) were used for comparisons as appropriate. Parametric tests were used when distribution was assumed to be normal and variance was assumed to be similar, but these were not formally tested. The sample sizes were chosen based on previous work [4, 30], as there was no pre-specified effect size.

## Abbreviations

ACC: Anterior cingulate cortex
ANOVA: Analysis of variance
dFC: Fear conditioning in a different context
Dox: Doxycycline
FC: Fear conditioning
GFP: Green fluorescent protein
Obs: Observation
PBS: Phosphate buffered saline
PFA: Paraformaldehyde
SEM: Standard error of the mean
tTA: tetracycline-transactivator
TTX: Tetrodotoxin
